# Pandemic risk characterisation of zoonotic influenza A viruses using the Tool for Influenza Pandemic Risk Assessment (TIPRA)

**DOI:** 10.1016/j.lanmic.2024.100973

**Published:** 2025-03

**Authors:** Reina Yamaji, Wenqing Zhang, Akiko Kamata, Cornelia Adlhoch, David E Swayne, Dmitriy Pereyaslov, Dayan Wang, Gabriele Neumann, Gounalan Pavade, Ian G Barr, Malik Peiris, Richard J Webby, Ron A M Fouchier, Sophie Von Dobschütz, Thomas Fabrizio, Yuelong Shu, Magdi Samaan

**Affiliations:** a Global Influenza Programme, Epidemic and Pandemic Preparedness and Prevention, WHO Emergency Programme, World Health Organization, Geneva, Switzerland; b The Food and Agriculture Organization of the UN (FAO), Rome, Italy; c Emerging Diseases and Zoonoses Unit, Department for Epidemic and Pandemic Preparedness and Prevention, World Health Organization, Geneva, Switzerland; d European Centre for Disease Prevention and Control, Solna, Sweden; e Birdflu Veterinarian, Watkinsville, GA, USA; f National Institute for Viral Disease Control and Prevention, China CDC, Changping District, Beijing, China; g Department of Pathobiological Sciences, School of Veterinary Medicine, University of Wisconsin-Madison, Madison, WI, USA; h World Organisation for Animal Health, Paris, France; i WHO Collaborating Centre for Reference and Research on Influenza, Victorian Infectious Diseases Reference Laboratory, Peter Doherty Institute for Infection and Immunity, Melbourne, VIC, Australia; j School of Public Health, Li Ka Shing Faculty of Medicine, The University of Hong Kong, Hong Kong Special Administrative Region, China; k Department of Infectious Diseases, St Jude Children's Research Hospital, Memphis, TN, USA; l Department of Viroscience, Erasmus Medical Centre, Rotterdam, Netherlands; m Institute of Pathogen Biology, Chinese Academy of Medical Sciences & Peking Union Medical College, Beijing, China

## Abstract

A systematic risk assessment approach is essential for evaluating the relative risk of influenza A viruses (IAVs) with pandemic potential. To achieve this, the Tool for Influenza Pandemic Risk Assessment (TIPRA) was developed under the Global Influenza Programme of WHO. Since its release in 2016 and update in 2020, TIPRA has been used to assess the pandemic risk of 11 zoonotic IAVs across ten evaluation rounds. Notably, A(H7N9), A(H9N2), and A(H5) clade 2.3.4.4 viruses were re-evaluated owing to changes in epidemiological characteristics or virus properties. A(H7N9) viruses had the highest relative risk at the time of assessment, highlighting the importance of continuous monitoring and reassessment as changes in epidemiological trends within animal and human populations can alter risk profiles. The knowledge gaps identified throughout the ten risk assessments should help to guide the efficient use of resources for future research, including surveillance. The TIPRA tool reflects the One Health approach and has proven crucial for closely monitoring virus dynamics in both human and non-human populations to enhance preparedness for potential IAV pandemics.

## Introduction

Influenza A viruses (IAVs) infect various host species and can cross species barriers through genetic mutation or reassortment. Recently, avian viruses with diverse haemagglutinins (HAs) have caused sporadic human infections, leading to high fatality rates, particularly with A(H5) and A(H7N9) infections.^1^ The WHO Global Influenza Programme (GIP), through the Global Influenza Surveillance and Response System, monitors the occurrence of zoonotic IAVs and unusual epidemiological characteristics in acute respiratory diseases. This monitoring includes detecting changes in viral properties as part of the One Health Initiative in collaboration with the Food and Agricultural Organization (FAO) and the World Organization for Animal Health (WOAH). To facilitate this effort, the GIP developed the Tool for Influenza Pandemic Risk Assessment (TIPRA), based on the Influenza Risk Assessment Tool (IRAT) developed by the WHO Collaborating Centre for Surveillance, Epidemiology & Control of Influenza at the US Centers for Disease Control and Prevention (CDC).^2^, ^3^, ^4^, ^5^ Influenza experts with diverse and multi-disciplinary expertise from WHO Collaborating Centres on Influenza, WHO regional Offices, FAO, WOAH, OFFLU (WOAH–FAO global network of expertise on animal influenza) reference laboratories, and other research institutes have participated in administering TIPRA to support interdisciplinary and comprehensive risk evaluation.

Here, we summarise the risk assessments of 11 zoonotic IAVs using TIPRA.

## Methods

### Overview

TIPRA supports hazard assessment of the overall public health risk of zoonotic IAVs by evaluating the likelihood of a virus acquiring human-to-human transmission capability and its potential public health impact in the event of such transmission. Each step of the risk assessment using the TIPRA is detailed in the TIPRA guidance—second edition.^3^

### Triggers for TIPRA usage

Triggers for performing a TIPRA evaluation include changes in mortality or incidence rates of human infections, circulation of novel or pathogenic viruses in animal populations, and changes in properties of circulating viruses. A consensus on the subtype and clade or lineage of viruses for TIPRA assessment is reached through consultation with public health professionals, technical experts, and other stakeholders.

### Risk elements and virus profile preparation

The GIP secretariat prepared a virus profile document summarising available data for each risk element in TIPRA. Ten risk elements were used to characterise the overall risk: four related to virus properties (receptor binding properties, genomic characteristics, transmission in animal models, and susceptibility to antiviral treatment), four related to human population attributes (human infection, disease severity, population immunity—likelihood, and population—immunity impact), and two related to virus ecology and epidemiology (geographical distribution in animals and infection in animals). Technical experts with expertise in at least one risk element were invited to participate in these assessments, review virus profiles, provide input, and share unpublished data when available.

### Scoring

The technical experts independently assigned four scores, with written explanations, for each risk element within their area of expertise, following the risk element guidance of TIPRA. These scores included a point estimate and both lower-bound and upper-bound estimates that they would accept from other technical experts for each risk element. These point estimates ranged from 1 to 10, where scores <4 indicated low risk, scores from 4 to 7·9 indicated moderate risk, and scores ≥8 indicated high risk. The fourth score, a confidence score, ranged from 1 (expert judgement only) to 5 (high confidence based on reliable datasets).

### Risk characterisation

Following expert scoring, the GIP secretariat conducted a preliminary analysis to calculate the overall risk scores using a multi-attribute additive model with rank-ordered centroid weights based on the importance of elements to likelihood or impact (tables 1, 2). During teleconferenced risk assessment exercises, technical experts discussed the reasoning for their scores, assessed potential biases in outliers, and identified knowledge gaps to develop recommendations for the virus assessed. After each teleconference, technical experts could revise their scores wherever necessary before finalising the assessment report.

**Table 1:** Risk scores and ranked weighting applied to 11 influenza A viruses for calculating the overall likelihood score

	Risk element weight	Subtype	A(H5)				A(H9N2)			A(H7N9)		A(H1N1) TRIG‡		A(H1Nx)
			A(H5N6)		A(H5Nx)	A(H5N1)								
		Clade or lineage	Clade 2.3.4.4	Clade 2.3.4.4	Clade 2.3.4.4	Clade 2.3.2.1c	Y280, G1 lineage	Y280 lineage	G1 lineage	NA		NA		1C lineage
		Assessment time	April 2016	November 2018	June 2021	December 2020	December 2016	March 2019	March 2019	September 2016	December 2017	April 2017		June 2022
Human infection	0·370		(4·20)∗	(4·50)	(3·85)	(4·20)	(4·67)	(4·86)	(2·91)	(6·13)	(5·85)	(4·14)		(4·73)
			1·556†	1·667	1·425	1·556	1·729	1·799	1·078	2·272	2·165	1·535		1·751
Population immunity - likelihood	0·228		(10·00)	(9·40)	(8·45)	(8·57)	(8·00)	(8·20)	(8·20)	(9·11)	(9·13)	Lower bound (4·71)	Upper bound (7·22)	(5·22)
			2·276	2·139	1·924	1·950	1·820	1·866	1·866	2·073	2·076	1·073	1·643	1·188
Transmission in animal models	0·156		(2·20)	(3·70)	(1·75)	(2·38)	(6·70)	(5·25)	(3·25)	(7·91)	(7·50)	(5·82)		(7·11)
			0·343	0·578	0·273	0·372	1·046	0·820	0·507	1·235	1·171	0·908		1·110
Receptor binding properties	0·109		(2·80)	(5·00)	(2·00)	(2·22)	(6·45)	(6·40)	(5·50)	(6·18)	(5·67)	(7·91)		(8·38)
			0·304	0·543	0·217	0·241	0·700	0·694	0·597	0·671	0·615	0·858		0·909
Genomic characteristics	0·073		(5·00)	(5·60)	(3·80)	(3·67)	(5·18)	(6·90)	(5·60)	(5·91)	(6·75)	(7·91)		(7·88)
			0·364	0·408	0·277	0·267	0·377	0·502	0·408	0·430	0·491	0·576		0·573
Infection in animals	0·044		(5·71)	(7·00)	(5·56)	(4·93)	(6·00)	(5·33)	(5·38)	(5·43)	(5·50)	(8·25)		(8·25)
			0·253	0·310	0·246	0·218	0·265	0·236	0·238	0·240	0·243	0·365		0·365
Geographical distribution in animals	0·020		(6·00)	(6·38)	(8·07)	(5·57)	(8·43)	(6·13)	(7·75)	(5·40)	(5·69)	(7·00)		(7·27)
			0·122	0·130	0·165	0·114	0·172	0·125	0·158	0·110	0·116	0·143		0·148
Overall score	1		5·218	5·773	4·526	4·718	6·110	6·042	4·851	7·031	6·878	5·457	6·028	6·045

NA=Not applicable.

∗ Numbers in parentheses indicate mean point estimate scores.

† Numbers without parentheses indicate mean point estimate scores *×* risk element weight. The risk element weights might not exactly sum up to 1 owing to rounding.

‡ Owing to scarce information on population immunity - likelihood at the time of risk assessment, some technical experts provided a range of scores.

**Table 2:** Risk scores and ranked weighting applied to 11 influenza A viruses for calculating the overall impact score

	Risk element weight	Subtype	A(H5)				A(H9N2)			A(H7N9)		A(H1N1) TRIG	A(H1Nx)
			A(H5N6)		A(H5Nx)	A(H5N1)							
		Clade or lineage	Clade 2.3.4.4	Clade 2.3.4.4	Clade 2.3.4.4b	Clade 2.3.2.1c	Y280, G1 lineage	Y280 lineage	G1 lineage	NA		NA	1C lineage
		Assessment time	April 2016	November 2018	June 2021	December 2020	December 2016	March 2019	March 2019	September 2016	December 2017	April 2017	June 2022
Disease severity	0·457		(8·63)∗	(7·89)	(2·75)	(8·17)	(4·00)	(3·60)	(2·88)	(8·14)	(8·22)	(2·00)	(4·22)
			3·939†	3·603	1·256	3·729	1·827	1·644	1·313	3·719	3·755	0·913	1·928
Population immunity - impact	0·257		(10·00)	(9·60)	(8·78)	(8·33)	(9·00)	(8·20)	(8·20)	(9·60)	(9·40)	(8·00)	(5·22)
			2·567	2·464	2·253	2·139	2·310	2·105	2·105	2·464	2·413	2·053	1·340
Susceptibility to antiviral treatment	0·157		(4·20)	(4·67)	(1·60)	(3·71)	(1·67)	(2·00)	(2·00)	(3·50)	(4·20)	(2·30)	(2·14)
			0·658	0·731	0·251	0·582	0·261	0·313	0·313	0·548	0·658	0·360	0·336
Genomic characteristics	0·090		(5·00)	(5·60)	(3·80)	(3·67)	(5·18)	(6·90)	(5·60)	(5·91)	(6·75)	(7·91)	(7·88)
			0·450	0·504	0·342	0·330	0·466	0·621	0·504	0·532	0·608	0·712	0·709
Receptor binding properties	0·040		(2·80)	(5·00)	(2·00)	(2·22)	(6·45)	(6·40)	(5·50)	(6·18)	(5·67)	(7·91)	(8·38)
			0·112	0·200	0·080	0·089	0·258	0·256	0·220	0·247	0·227	0·316	0·335
Total	1		7·725	7·502	4·181	6·869	5·122	4·939	4·455	7·510	7·660	4·355	4·648

NA= Not applicable.

∗ Numbers in parentheses indicate mean point estimate scores.

† Numbers without parentheses indicate mean point estimate scores × risk element weight. The risk element weights might not exactly sum up to 1 owing to rounding.

### Measure of uncertainty

The risk characterisation of TIPRA relies on qualitative expert scoring based on the virus profile and compilation of available data at the time of risk assessment. Since the assessed groups of viruses could show genetic and phenotypic differences, and the data available might be insufficient for definitive conclusions, experts might provide range estimates (ie, the lower and upper boundaries of point estimate scores) to capture the degree of uncertainty in point estimates. A confidence score, which typically reflects the quality and quantity of available data for each risk element in the context of the experience and expertise of each technical expert, was provided along with these estimates.

## Results

### Risk of A(H5) viruses

Since the first human outbreak caused by A/Goose/Guangdong/1/96-Eurasian lineage A(H5N1) viruses in Hong Kong Special Administrative Region, China, in 1997, these viruses have evolved into multiple HA phylogenetic clades.^6^^,^^7^ Owing to their distinct epidemiological and virological properties, different clades have been independently assessed using TIPRA.

### Clade 2.3.4.4 A(H5N6) virus in 2016

Clade 2.3.4.4 A(H5) viruses underwent a series of reassortment events, leading to the creation of novel subtype HA-NA (neuraminidase) combinations.^8^ Furthermore, with distinct evolutionary pathways, the HA gene of clade 2.3.4.4 viruses diversified into further genetic subgroups (subgroups a-h), some of which were geographically defined.^9^

**Figure 1:**
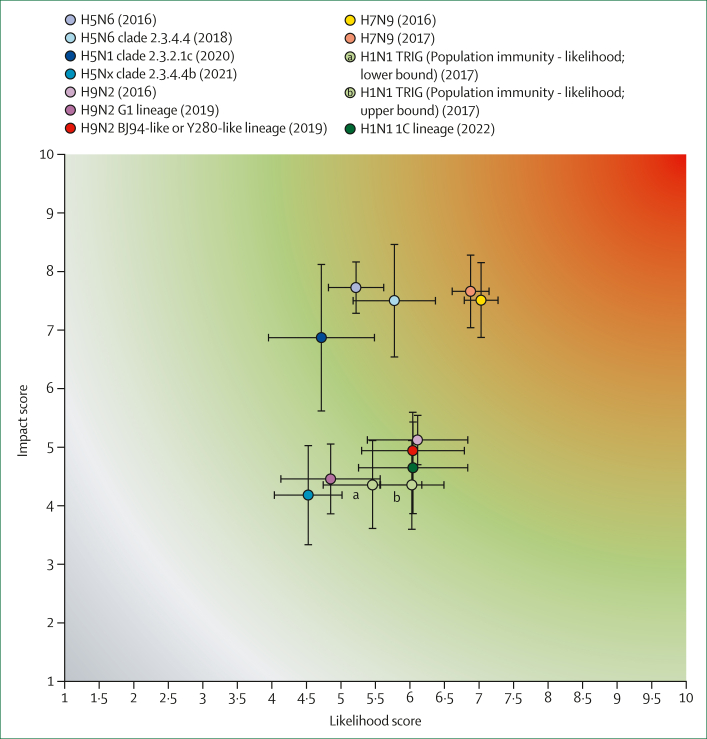
Risk map of the Tool for Influenza Pandemic Risk Assessment results Risk map depicting the overall virus risk, with the likelihood of the virus plotted on the x-axis and impact risk scores on the y-axis.

Beginning in 2013, reassortant A(H5N6) clade 2.3.4.4 viruses caused multiple outbreaks in poultry in China, Viet Nam, and Laos.^10^ By February, 2016, ten human infections by A(H5N6) clade 2.3.4.4 viruses, including six fatal cases, had been detected in China.^1^ Owing to the wide geographical range of A(H5) viruses and sporadic human A(H5N6) infections, the first TIPRA risk assessment was conducted in April, 2016. A high case-fatality rate (CFR) and low population immunity contributed to an overall impact score of 7·725, the highest among all assessed viruses (table 2, figure 1). A(H5N6) viruses did not transmit via respiratory droplets between ferrets in laboratory settings; however, some transmission was observed between guinea pigs. No biophysical assay data on receptor binding properties were available during risk assessment. These factors contributed to the overall pandemic likelihood score of 5·218 for A(H5N6) viruses (table 1, figure 1).

### Clade 2.3.4.4 A(H5N6) virus in 2018

As of November, 2018, the number of human cases of A(H5N6) in China had increased to 22.^1^ Additionally, the geographical range of these viruses had expanded to bird populations in the Middle East and Africa.^11^, ^12^, ^13^, ^14^, ^15^, ^16^ This geographical expansion and the rise in human infections triggered the second TIPRA exercise on this virus group in November, 2018. The overall impact score was 7·502, with no substantial change from the first assessment (table 2, figure 1). Since the first assessment, additional data had been generated indicating that some viruses had dual receptor binding to both human-type and avian-type receptors,^17^, ^18^, ^19^, ^20^, ^21^, ^22^ which increased the mean point estimate for receptor binding properties from 2·80 to 5·00 (table 1). Although transmissibility of the viruses via respiratory droplets in ferrets had not been reported, new data showed stable transmissibility via direct contact.^23^, ^24^, ^25^ Consequently, the mean point estimate scores for transmission in animal models increased from 2·20 to 3·70 (table 1). The overall likelihood score increased slightly to 5·773 (table 1, figure 1). Given the high variability in scores for human infection and transmission in animal models, which are heavily weighted in calculating the overall likelihood score, the error bars overlapped with those of the clade 2.3.4.4 A(H5N6) virus in 2016 (figure 1).

### Clade 2.3.2.1c A(H5N1) virus in 2020

From 2007 to 2010, clade 2.3.2.1 A(H5N1) viruses became prevalent in China and southeast Asia, later spreading to eastern Asia and Russia. This spread was accompanied by HA diversification and led to the designation of three further clades: 2.3.2.1a, b, and c.^26^, ^27^, ^28^ From 2015, clade 2.3.2.1c A(H5N1) viruses had been detected in multiple continents. However, by 2017, they were primarily active in Africa and Asia. Owing to the widespread distribution of the virus in poultry and six reported human cases, a TIPRA exercise was conducted in December, 2020. The high number of human infections and lack of population immunity resulted in relatively high scores in the respective categories; however, these scores were offset by the lack of adaptation to mammalian hosts^21^^,^^29^, ^30^, ^31^ and a smaller geographical range in poultry compared with that during earlier years. The overall likelihood score was 4·718 (table 1, figure 1). Variation in the susceptibility to antiviral treatment score was primarily driven by one study showing reduced susceptibility to zanamivir and oseltamivir in several viruses isolated from wild birds;^32^ however, few clade 2.3.2.1c viruses possessed amino acid substitutions associated with reduced susceptibility to neuraminidase (NA) inhibitors (appendix p 3). The overall impact score for clade 2.3.2.1c A(H5N1) viruses was 6·869 (table 2, figure 1).

### Clade 2.3.4.4b A(H5Nx) virus in 2021

Clade 2.3.4.4b viruses emerged as the dominant form of the subtype in 2020.^33^ Notably, A(H5N1) clade 2.3.4.4b viruses spread to several countries in Europe, Africa, and Asia during 2020–21.^34^, ^35^, ^36^, ^37^ In December, 2020, seven human cases of A(H5N8) clade 2.3.4.4b viruses were identified in Russia.^38^^,^^39^ As a result of seven reported human cases and increased geographical spread, a TIPRA exercise was conducted in June, 2021. Given that all detected human cases were asymptomatic, the overall impact score was marked at 4·181, which was much lower than for all other H5 viruses that have been scored (table 2, figure 1). The increased geographical spread of this virus clade in avian populations^13^^,^^40^ and low population immunity resulted in high scores for respective risk elements; however, these scores were partly offset by the lack of virus adaptation to human or mammalian hosts. The overall likelihood score was 4·526 (table 1, figure 1), which was similar to the 2020 H5N1 score. Since the risk assessment, following the spread of clade 2.3.4.4b A(H5N1) viruses across multiple continents, the virus reached North America by late 2021 and South America by autumn 2022. Since October, 2021, A(H5N1) has overtaken A(H5N8) as the major A(H5) virus, causing bird outbreaks, accompanied by spillover infections in mammals reported in Asia, Europe, North America, and South America.^41^, ^42^, ^43^ Additional human infections have been reported in China, Nigeria, the UK, the USA, and Spain.^38^^,^^44^, ^45^, ^46^, ^47^ Furthermore, clade 2.3.4.4b A(H5N1) was detected in dairy cattle in the USA for the first time, resulting in three human infections as of June, 2024. Consequently, a risk assessment for clade 2.3.4.4b A(H5N1) genotype B3.13 viruses is being planned.

### Risk of A(H9N2) virus

A(H9N2) Eurasian lineage viruses are currently the most widespread low-pathogenicity avian influenza (LPAI) viruses in poultry populations globally. From April, 1999 to December, 2016, two of the three sub-lineages of A(H9N2) Eurasian lineage viruses—BJ94-like or Y280-like and G1—caused 37 recorded human infections.^48^ These dominant A(H9N2) lineages reportedly contributed gene segments to other zoonotic influenza viruses, including A(H5Nx), A(H7N9), and A(H10N8). A(H9N2) infections in poultry are often undetected owing to their low virulence, resulting in their unnoticed spread among the avian population and frequent reassortment.^48^^,^^49^

### A(H9N2) viruses in 2016

For the first assessment in 2016, A(H9N2) Eurasian lineage viruses were assessed as a single group. The overall likelihood and impact scores were 6·110 and 5·122, respectively (tables 1, 2, figure 1). These results reflected a consensus among technical experts regarding the generally mild disease course in humans and the lack of human infection clusters. Technical experts also agreed on other properties of A(H9N2) viruses: some viruses preferentially bound to human α2,6-linked sialic acid, whereas others retained the ability to bind to avian-type receptors,^50^^,^^51^ and some were transmissible or highly transmissible in ferrets via respiratory droplets.^52^, ^53^, ^54^, ^55^

### BJ94-like or Y280-like lineage and G1-lineage A(H9N2) viruses in 2019

**Figure 2:**
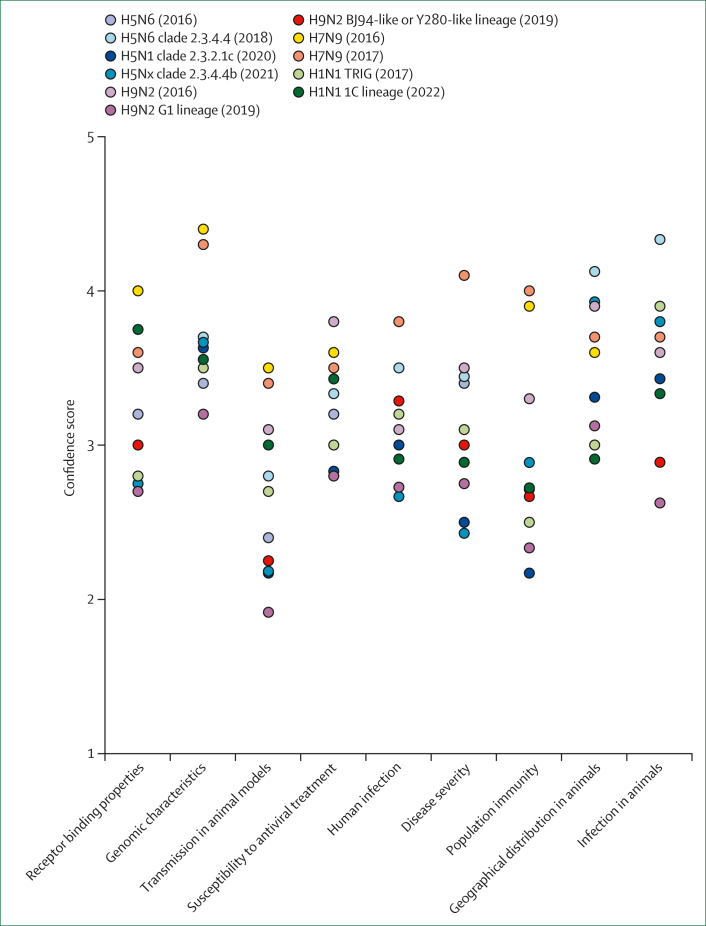
Distribution of the mean confidence scores for all viruses assessed up to August, 2022 Confidence scores for each risk element, representing the breadth and quality of evidence specific to each element, are shown.

The 2016 assessment highlighted the genetic diversity within A(H9N2) viruses and the potential for varying risks across specific lineages. Consequently, the BJ94-like or Y280-like and G1 lineages were assessed separately during the second assessment in March, 2019. As predicted in the 2016 exercise, differences in scoring between these two lineages were apparent, particularly in four risk elements: human infection, geographical distribution in animals, transmission in animal models, and genomic characteristics (table 1), which showed differences in the average point estimate scores (by greater than one point). The 16 human cases of A(H9N2) reported in China since December, 2016 were all caused by the BJ94-like or Y280-like lineage, despite the broader geographical distribution of the G1 lineage in poultry across Asia, Africa, and Europe.^56^ No additional ferret transmission studies had been conducted since the first assessment, and variability in interpreting data from miniature pig studies resulted in a wide range of point estimate scores and low confidence scores for transmission in animal models among technical experts (figure 2, appendix p 3).^57^^,^^58^ The degree to which serological cross-reactivity in individuals born before 1968 confers protection against A(H9N2) infection remains unknown; therefore, technical experts agreed on a higher-risk category score for population immunity.^59^^,^^60^ The overall likelihood and impact scores were 6·042 and 4·939 for the BJ94-like or Y280-like lineage, respectively, and 4·851 and 4·455 for the G1 lineage of the A(H9N2) virus, respectively (Table 1, Table 2, figure 1).

In April, 2022, the first A(H3N8) Eurasian lineage human infections were reported in China.^61^ Phylogenetic analysis of the full genome sequences of two laboratory-confirmed human cases and newly isolated A(H3N8) viruses from birds in Hong Kong Special Administrative Region revealed that their six internal genes were derived from a dominant genotype of BJ94-like or Y280-like lineage A(H9N2) viruses in China.^62^, ^63^, ^64^ Considering the direct and indirect pandemic risks associated with A(H9N2) viruses, changes in their biological properties might trigger further risk assessments.

### A(H7N9) virus in 2016

Following the first report of three human cases of A(H7N9) Eurasian lineage infection in China in March, 2013,^65^ LPAI A(H7N9) viruses caused four epidemic waves in humans in China, with 798 human cases reported as of September, 2016, including 320 deaths, reflecting a 40% CFR among reported cases.^1^ At that time, the virus was asymptomatic in poultry. The high number of human cases and observed pathogenicity triggered the first A(H7N9) TIPRA assessment in September, 2016. Most available data were generated from two A(H7N9) human virus isolates, A/Anhui/1/2013 and A/Shanghai/1/2013. These viruses replicated efficiently in human respiratory cells,^66^ had dual receptor binding specificity,^67^^,^^68^ and were capable of respiratory droplet transmission in ferret models.^69^, ^70^, ^71^ The overall likelihood score was 7·031, the highest among all viruses assessed to date (table 1, figure 1). A high CFR and low population immunity also contributed to a high overall impact score of 7·510 (table 2, figure 1).

### A(H7N9) virus in 2017

Between the October, 2016 A(H7N9) TIPRA exercise and September, 2017, the fifth wave of human infections by A(H7N9) occurred, resulting in 767 laboratory-confirmed cases and a 38% CFR among recorded cases. 31 imported A(H7N9) human cases from China were also reported in various countries.^1^ During this period, high-pathogenicity avian influenza (HPAI) forms of the A(H7N9) virus emerged for the first time in poultry, accounting for 28 human cases.^72^ The evolving A(H7N9) situation led to another TIPRA exercise in December, 2017. The properties of the HPAI A(H7N9) virus A/Guangdong/17SF003/2016, used for viral characterisation in many studies,^73^, ^74^, ^75^ were similar to the earlier LPAI A(H7N9) viruses. The updated likelihood score of 6·878 was similar to that of the first assessment (table 1, figure 1). No difference in CFR was observed between viruses from the fifth wave and earlier waves. However, the highly pathogenic form of the virus in poultry, responsible for some human cases in the fifth wave, more frequently carried the R292K mutation in the NA protein, which increases resistance to some NA-inhibitor-based antiviral drugs.^76^ This finding led to an increase in the mean point estimate score for susceptibility to antiviral treatment from 3·50 in the first assessment to 4·20 in the second. Consequently, the overall impact score reached 7·660, the second highest among all viruses assessed to date (table 2, figure 1).

In China, in addition to market closures, disinfection, and depopulation efforts, the implementation of a massive poultry vaccination campaign in September, 2017 using an inactivated A(H5)+A(H7) bivalent vaccine coincided with a considerable decline in the number of reported human cases.^77^, ^78^, ^79^ The H5+H7 vaccine continues to be updated based on circulating field strains and administered to domestic poultry in China.^80^^,^^81^ Although only sporadic human cases of A(H7N9) have been reported since 2018, close monitoring of changes in virus properties is warranted.

### Overview of swine A(H1) viruses

The three subtypes of influenza viruses most identified in pigs are A(H1N1), A(H1N2), and A(H3N2). The biological characteristics of the specific viruses that circulate worldwide vary by geographical region, with ample evidence for bi-directional transmission between pigs and humans. To date, three major lineages of swine A(H1) viruses have been recognised—1A (classical swine lineage), 1B (human seasonal lineage), and 1C (Eurasian avian lineage).^82^

### Swine A(H1N1) 1A triple-reassortant virus in 2017

In the late 1990s, swine A(H1) viruses, originating from the virus responsible for the 1918 Spanish influenza pandemic and currently classified into the 1A lineage, reassorted with triple-reassortant H3N2 virus lineage. Infections caused by these A(H1) viruses possessing a triple reassortment internal gene cassette became enzootic in pigs in North America.^83^^,^^84^ By February, 2009, six human infections with these A(H1N1) viruses were reported in the USA. Driven by the fact that these viruses contributed six of the eight gene segments to the A(H1N1)pdm09 strain,^85^ a historical TIPRA assessment using information available before the 2009 A(H1N1) influenza pandemic was conducted in April, 2017, as an exercise. Due to the scarcity of seroprevalence data among the general population,^86^ some technical experts provided a range of scores from 4 to 7 for population immunity - likelihood (table 1). Consequently, the overall likelihood score ranged from 5·457 to 6·028 (table 1, figure 1). The presence of molecular and phenotypic markers of mammalian adaptation led to the highest mean point estimate scores in genomic characteristics and receptor binding properties among all the viruses assessed.^87^^,^^88^ The lack of experimental ferret transmission data resulted in experts assigning a score of 5·82 for the transmission in animal models risk element, categorising it as a moderate risk (table 1). No hospitalisations occurred among the six reported cases, and because of no human-to-human transmission, the mean point estimate for disease severity was low, which contributed to the overall impact score of 4·355 (table 2, figure 1). This exercise provided proof of principle assessment of the validity of the TIPRA approach by its retrospective application to a real pandemic threat.

### Swine A(H1) 1C (Eurasian avian-like) virus in 2022

In the late 1970s, an avian A(H1) virus was reported in pigs in Europe, with subsequent spread to Asia.^89^^,^^90^ Between 2017 and 2022, 18 human cases of infection with swine influenza A(H1) 1C lineage viruses were reported. Due to its geographical dispersal in the pig population, continual reporting of human cases, and evolving gene constellations, risk assessment was conducted using TIPRA in June, 2022. The overall likelihood score was 6·045, and the overall impact score was 4·648 (table 2, figure 1). Reflecting mammalian adaptation of the viruses, especially genotype 4, which consisted of internal genes from the human A(H1N1)pdm09 and North American triple reassortment internal gene, transmission in animal models, receptor binding properties, and genomic characteristics risk elements had mean point estimate scores greater than 7, with technical expert consensus (table 1).^91^, ^92^, ^93^, ^94^ There was less consensus on population immunity elements, with different interpretations of protective efficacies of observed serological crossreactivities in humans.^95^ Thus, the distribution of individual scores ranged from 3 to 9 for population immunity - likelihood, leading to a mean point estimate of 5·22 (appendix p 5). Thus, although swine A(H1) 1C lineage viruses currently circulating worldwide do not have the ability for human-to-human transmission,^96^ surveillance of pig populations should be strengthened for the early detection of the emerging traits of the viruses.

### Knowledge gaps identified during the TIPRA assessments

Confidence in scoring the risk elements and final risk scores of TIPRA relies on the amount, depth, and quality of the information available at the time of the risk assessment exercise and the experience and expertise of the technical experts. Identifying knowledge gaps to aid in prioritising resources for further research and surveillance is one of TIPRA's important steps and objectives. A(H7N9) viruses had high confidence scores among the viruses assessed, whereas A(H9N2) viruses showed low confidence scores, which was reflective of the paucity of data for virus characterisation (figure 2). The necessity of more phenotypic assays was flagged in two risk elements defining virus properties: receptor binding properties and susceptibility to antiviral treatment. In-vitro studies such as solid-phase binding assays or glycan arrays are the first proxy for assessing receptor binding properties; however, the glycans used in these in-vitro platforms might not be identical to the glycans expressed by the respective human or animal tissues. Thus, studies with ex-vivo tissues could provide valuable information for risk assessment. Technical experts cautioned relying solely on sequencing data for inferring susceptibility to antiviral treatment because of the unrecognised influence of other amino acid mutations on biological properties. Viral sequence analysis from currently under-sampled geographical locations and species, along with the timely sharing of these data, could improve the risk assessment of genome characteristics. Discussion of the transmission in animal models recognised challenges in the interpretation of results due to the variability of experimental study designs, especially those in ferret transmission studies.^97^^,^^98^ Establishment of alternative transmission models (such as Syrian hamsters or guinea pigs) could thus improve risk assessments. Data on seroprevalence targeted to the general population were scarce, which made obtaining high confidence scores for population immunity difficult. Researchers have showed that assessing population immunity by age-stratified seroprevalence by calculating the impact of population immunity on the basic reproduction number (R_0_) impedes the spread of the virus.^64^^,^^95^ Technical experts were in agreement that more surveillance data were needed from regions beyond the geographical areas where the virus was endemic. Most technical experts also suggested that the challenge of scoring the risk elements for swine influenza viruses and avian A(H9N2) viruses, both of which are not reportable to the WOAH, resulted in low confidence in their scores.

## Discussion

In this Review, we have summarised the rationale for selecting viruses for risk assessment using TIPRA, risk characterisation results, and recommendations for research and surveillance to bridge crucial knowledge gaps of IAVs. TIPRA is based on the collective work of a broad spectrum of global stakeholders. Repeated risk assessments of influenza A(H7N9), A(H9N2), and A(H5Nx) viruses helped to track the chronological change in the pandemic risk level of the virus that might necessitate the reformulation of countermeasures. Notably, A(H7N9) viruses have not been reassessed since a substantial drop in their prevalence in 2018 in poultry in China after the implementation of an updated poultry vaccine. From the risk assessments using TIPRA, A(H5N1), A(H5N6), and A(H7N9) were found to have the highest overall impact scores because of a high CFR among recorded cases.

TIPRA is not a tool for predicting the subtypes of zoonotic IAV that will cause the next pandemic; its risk characterisation should be interpreted comprehensively with caution. The overall risk scores represent risk evaluation based on data available during the exercise, which could change with the availability of new evidence-based data. Additionally, TIPRA focuses on essential virological, epidemiological, and clinical information as part of the hazard assessment. Thus, the outputs of TIPRA should be contextualised based on exposure and context components, which can be assessed with other tools such as WHO's Rapid Risk Assessment of Acute Public Health Events (2012).^99^ A key element of TIPRA is its consensus-based assessment, minimising the subjectivity by technical experts with extensive expertise to cover all risk elements sufficiently. Furthermore, the initial teleconference following scoring by a technical expert provided an opportunity to discuss the reasonings for individual scores and re-assess the validity of individual scores. Score discrepancies, resulting from scarce data and varying interpretations by experts, were accepted and valued as they reflect the circumstances present during the time of the risk assessment (appendix pp 1–6). The risk map (figure 1) symbolises the middle ground of diverse opinions among experts, and attention should be paid to the range of scores and the associated confidence scores (figure 2). The error bars of the overall risk scores of likelihood and impact indicated the variations in expert scoring (figure 1).

Comparing risk assessment results with TIPRA and IRAT also provides noteworthy insights for their comprehensive understanding.^2^ Despite methodical differences,^2^^,^^3^ six (85·7%) of seven viruses were assessed similarly with both tools, except for swine A(H1) 1C virus (appendix p 7).

In general, the ten representative elements used in the risk stratification guidance of TIPRA are considered the most relevant and realistic set of factors that have successfully served as assessment criteria as per our latest understanding of IAVs. At present, genomic sequencing data of the viruses are unable to predict their phenotypes because of the diverse gene constellations of IAV. Therefore, the dataset of the findings of in-vivo and in-vitro experiments generated by multiple laboratories worldwide on each zoonotic threat should be collected. Timely sharing of IAVs should also be highly encouraged to facilitate full risk assessments.

TIPRA scores have the potential to inform and affect various applied science and regulatory measures. Furthermore, TIPRA assessments can direct research funding to areas at high risk of IAV infections and enhance targeted surveillance efforts, ultimately contributing to better preparedness and response strategies against potential influenza pandemics. To align global health initiatives and optimise resource allocation, the use of TIPRA and updating its risk stratification guide should continue as part of the ongoing global concerted efforts to mitigate the potential pandemic risk of IAVs.

## Declaration of interests

GN is a Co-Founder of FluGen. All other authors declare no competing interests.
