# Supplementary material for: Pandemic risk characterisation of zoonotic influenza A viruses using the Tool for Influenza Pandemic Risk Assessment (TIPRA)
Source: Lancet Microbe. 2025 Mar;6(3):None. doi: 10.1016/j.lanmic.2024.100973 (PMC11876097; doi:10.1016/j.lanmic.2024.100973)
Supplement: Supplementary appendix [file mmc1.pdf]

THE LANCET
Microbe

Supplementary appendix

This appendix formed part of the original submission and has been peer reviewed. We post it as supplied by the authors.

Supplement to: Yamaji R, Zhang W, Kamata A, et al. Pandemic risk characterisation of zoonotic influenza A viruses using the Tool for Influenza Pandemic Risk Assessment (TIPRA). *Lancet Microbe* 2025. https://doi.org/10.1016/j.lanmic.2024.100973

Table of Contents

Supplementary Figure 1a: Receptor Binding Properties 2
Supplementary Figure 1b: Genomic Characteristics 2
Supplementary Figure 1c: Transmission in Animal Models 3
Supplementary Figure 1d: Susceptibility to Antiviral Treatment 3
Supplementary Figure 1e: Human Infection 4
Supplementary Figure 1f: Disease Severity 4
Supplementary Figure 1g: Population Immunity - Likelihood 5
Supplementary Figure 1h: Population Immunity – Impact 5
Supplementary Figure 1i: Geographic Distribution in Animals 6
Supplementary Figure 1j: Infection in Animals 6
Supplemental Table 1: Comparison of Risk Assessment Results of influenza A viruses assessed with TIPRA and IRAT 8

Supplementary Figure 1a: Receptor Binding Properties

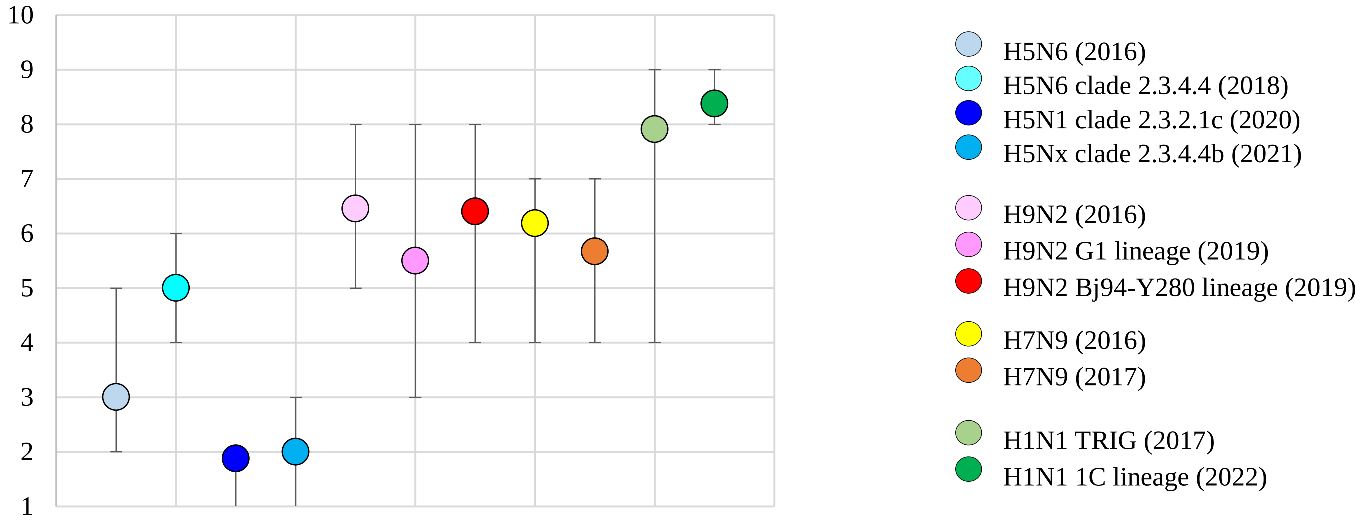

Mean point estimate scores and the range of point estimate values scored by technical experts

Supplementary Figure 1b: Genomic Characteristics

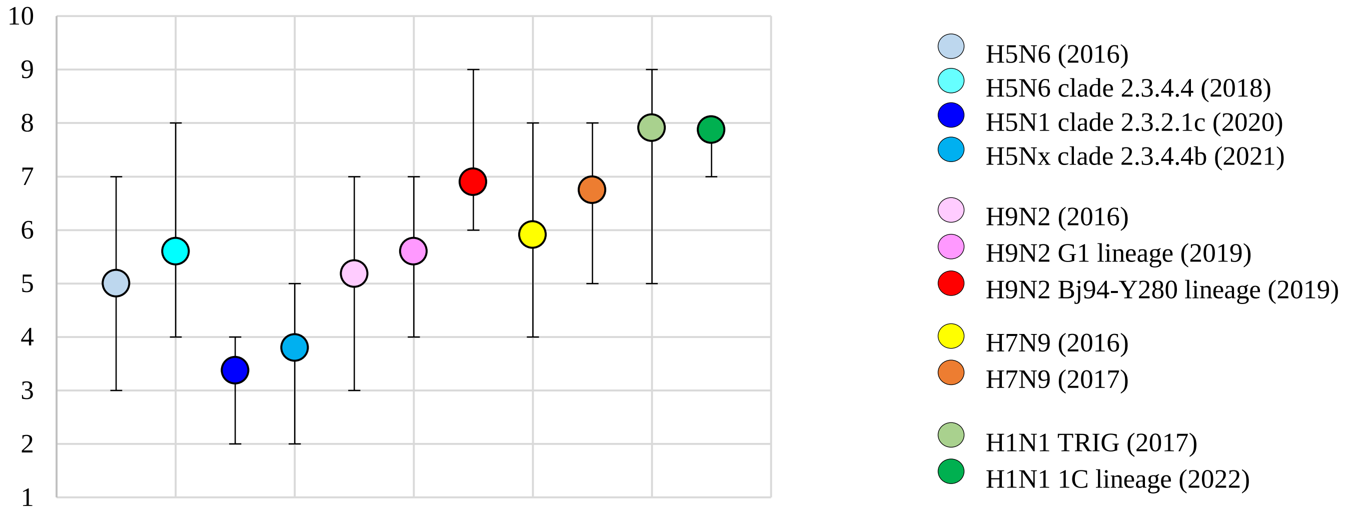

Mean point estimate scores and the range of point estimate values scored by technical experts

Supplementary Figure 1c: Transmission in Animal Models

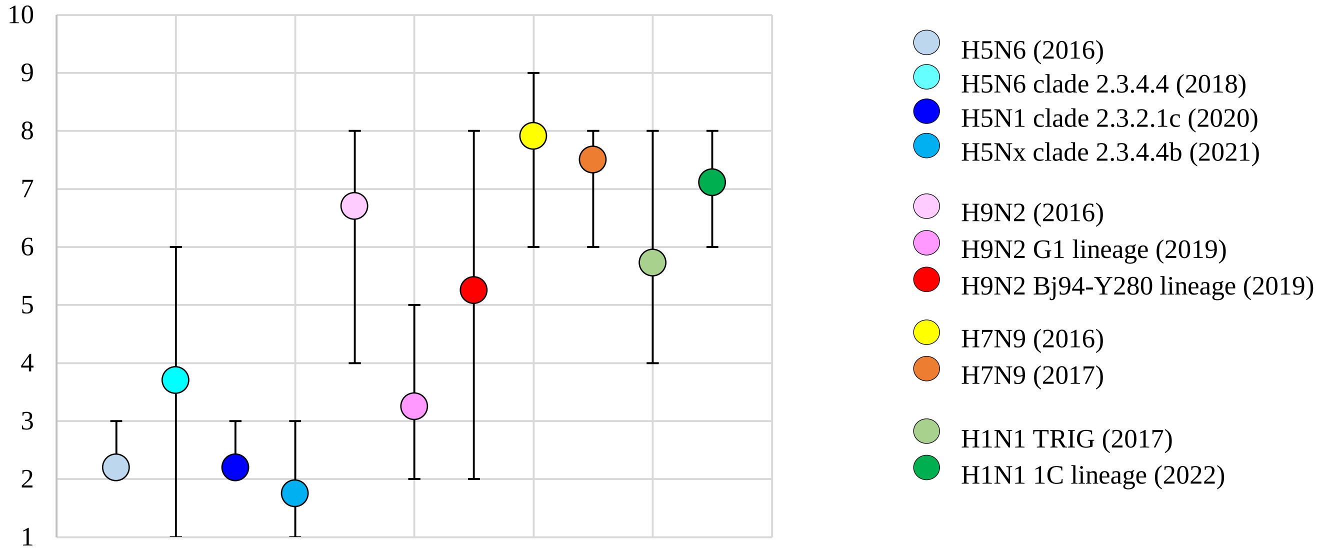

Mean point estimate scores and the range of point estimate values scored by technical experts

Supplementary Figure 1d: Susceptibility to Antiviral Treatment

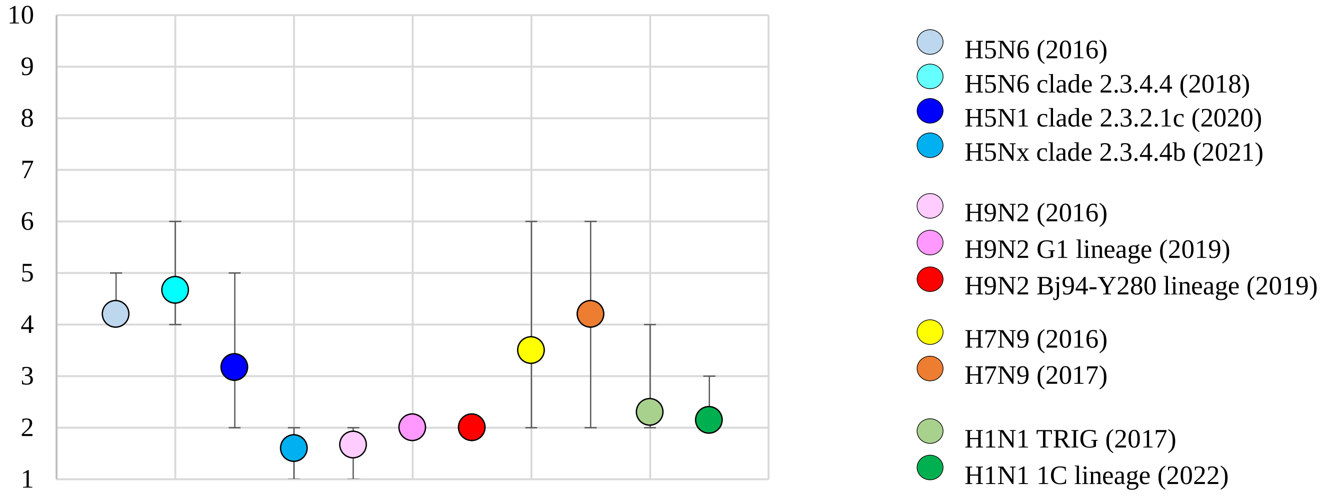

Mean point estimate scores and the range of point estimate values scored by technical experts

Supplementary Figure 1e: Human Infection

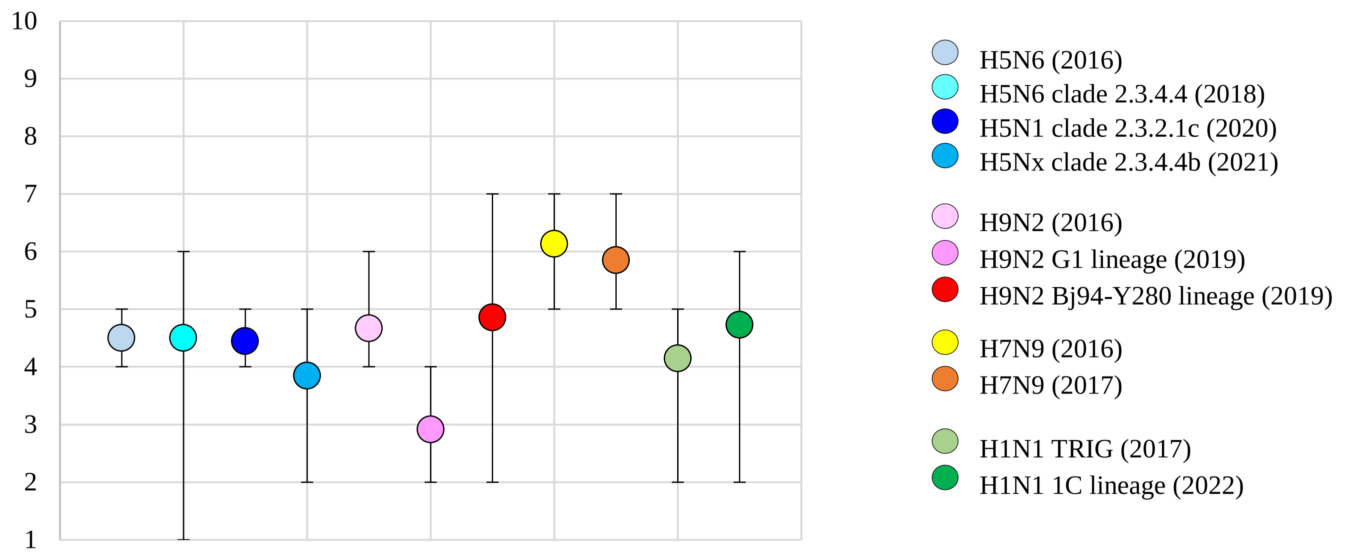

Mean point estimate scores and the range of point estimate values scored by technical experts

Supplementary Figure 1f: Disease Severity

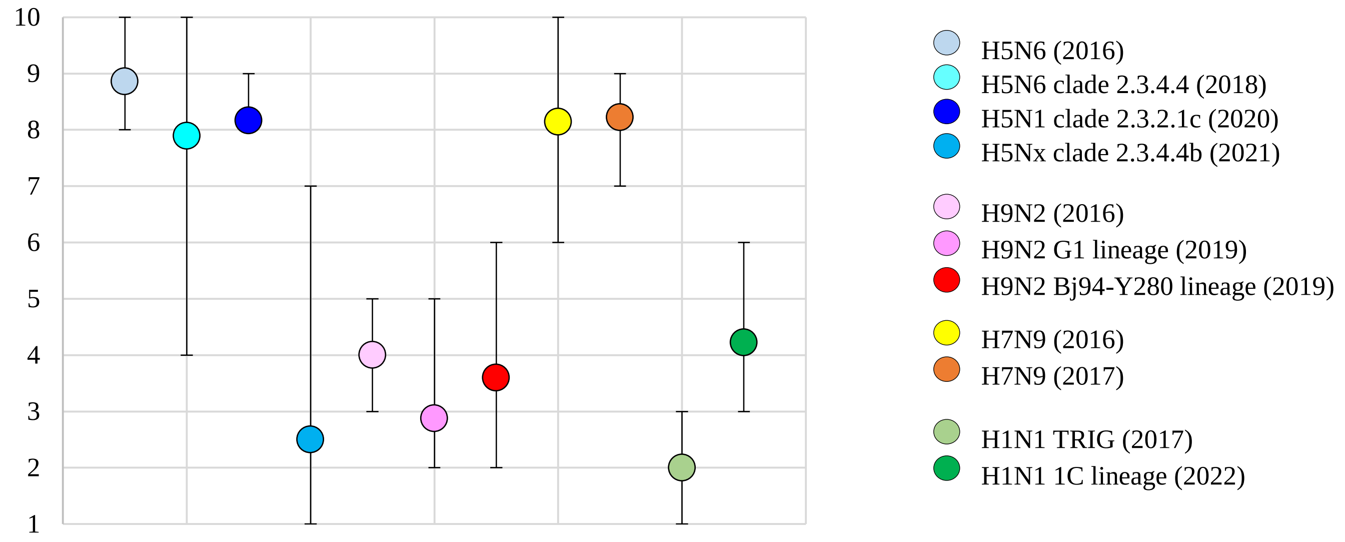

Mean point estimate scores and the range of point estimate values scored by technical experts

Supplementary Figure 1g: Population Immunity - Likelihood

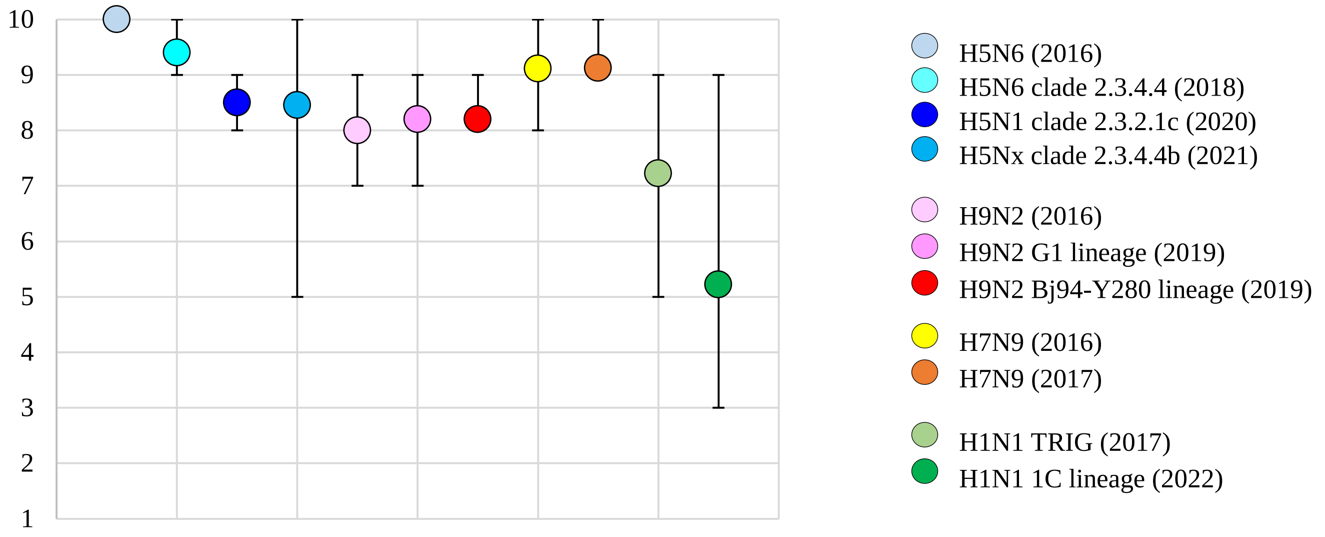

Mean point estimate scores and the range of point estimate values scored by technical experts

Supplementary Figure 1h: Population Immunity – Impact

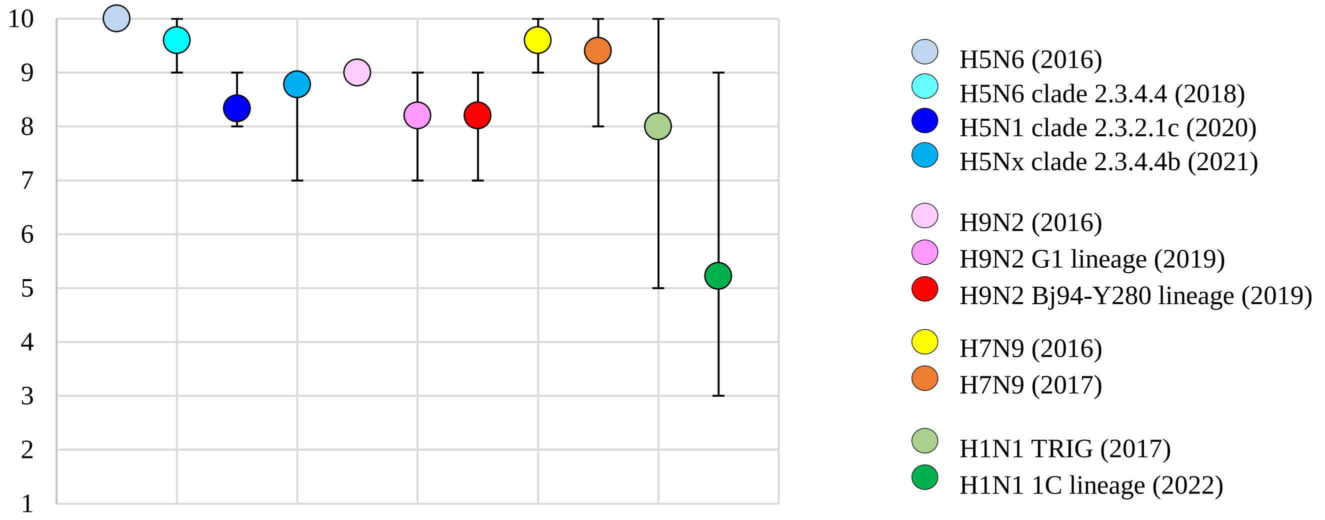

Mean point estimate scores and the range of point estimate values scored by technical experts

Supplementary Figure 1i: Geographic Distribution in Animals

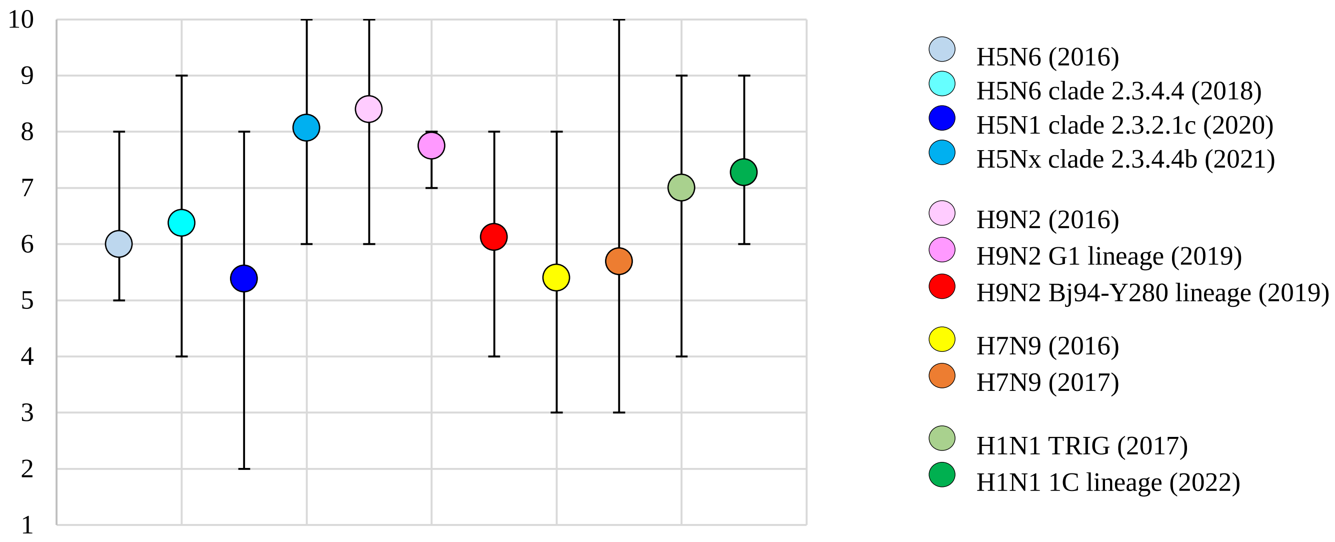

Mean point estimate scores and the range of point estimate values scored by technical experts

Supplementary Figure 1j: Infection in Animals

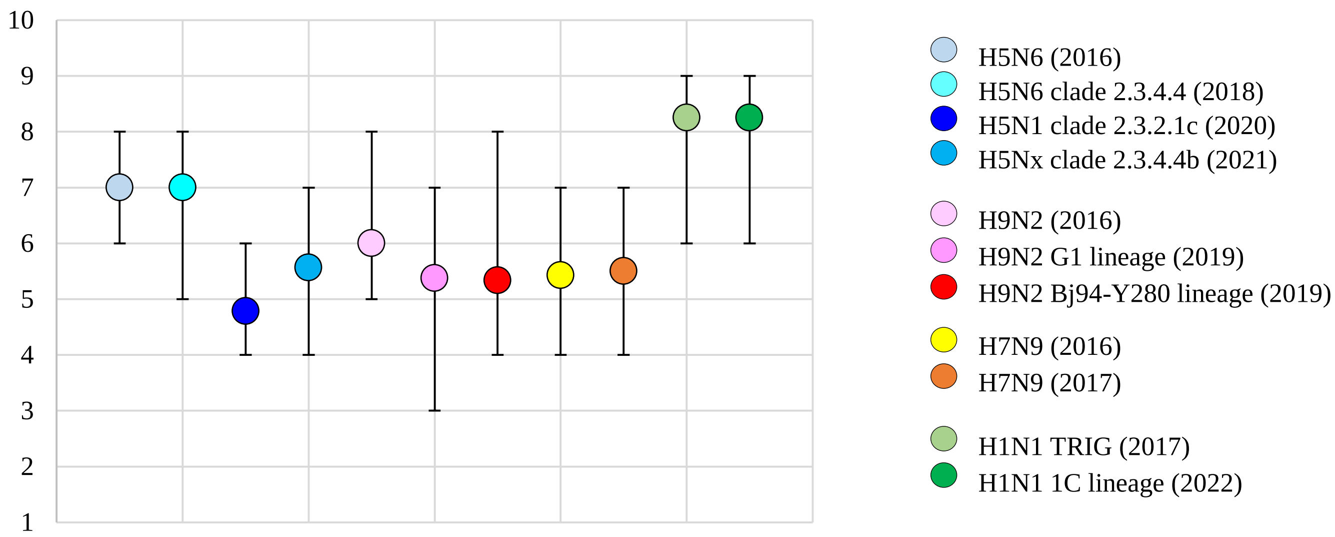

Mean point estimate scores and the range of point estimate values scored by technical experts

Supplemental Table 1: Comparison of Risk Assessment Results of influenza A viruses assessed with TIPRA and IRAT

Serial No·	Tool to Assess	Virus	Prototype Virus	Assessment Date	Likelihood/ Emergence Score	Difference in Likelihood/Emergence Scores between TIPRA and IRAT	Impact Score	Difference in Impact Scores between TIPRA and IRAT
1	TIPRA	Clade 2.3.4.4 A(H5N6)	NA	Apr 2016	5·22		7·73	
	IRAT	A(H5N6)	A/Yunnan/14564/2015	Apr 2016	5·50	0·28	6·60	1·13
2	TIPRA	Clade 2.3.4.4b A(H5Nx)	NA	Jun 2021	4·53		4·18	
	IRAT	Clade 2.3.4.4b A(H5N8)	A/Astrakhan/3212/2020	Mar 2021	4·60	0·07	5·20	1·02
3	TIPRA	BJ94/Y280 lineage A(H9N2)	NA	Mar 2019	6·04		4·94	
	IRAT	BJ94/Y280 lineage A(H9N2)	A/Anhui-Lujian/13/2018	Jul 2019	6·20	0·16	5·90	0·96
4	TIPRA	G1 lineage A(H9N2)	NA	Mar 2023	4·85		4·46	
	IRAT	G1 lineage A(H9N2)	A/Bangladesh/0994/2011	Feb 2014	5·60	0·75	5·40	0·94
5	TIPRA	A(H7N9)	NA	Sep 2016	7·03		7·51	
	IRAT	A(H7N9)	A/Shanghai/02/2013	Apr 2016	6·40	0·63	7·20	0·31
6	TIPRA	A(H7N9)	NA	Dec 2017	6·87		7·66	
	IRAT	A(H7N9)	A/Hong Kong/125/2017	May 2017	6·50	0·37	7·50	0·16
7	TIPRA	swine A(H1Nx) 1C	NA	Jun 2022	6·04		4·65	
	IRAT	swine A(H1N1) 1C, Genotype 4	A/swine/Shandong/1207/2016	Jul 2020	7·50	1·46	6·90	2·25

NA denotes not applicable. No specific strain was selected for risk assessment with TIPRA, but the clade or subclade of viruses were selected.
